# Glucagon kinetics assessed by mathematical modelling during oral glucose administration in people spanning from normal glucose tolerance to type 2 diabetes

**DOI:** 10.3389/fendo.2024.1376530

**Published:** 2024-04-12

**Authors:** Francesco Andreozzi, Elettra Mancuso, Mariangela Rubino, Benedetta Salvatori, Micaela Morettini, Giuseppe Monea, Christian Göbl, Gaia Chiara Mannino, Andrea Tura

**Affiliations:** ^1^ Department of Medical and Surgical Sciences, Magna Graecia University of Catanzaro, Catanzaro, Italy; ^2^ CNR Institute of Neuroscience, Padova, Italy; ^3^ Department of Information Engineering, Università Politecnica delle Marche, Ancona, Italy; ^4^ Department of Obstetrics and Gynaecology, Medical University of Vienna, Vienna, Austria; ^5^ Department of Obstetrics and Gynaecology, Medical University of Graz, Graz, Austria

**Keywords:** glucagon, alpha-cell insulin sensitivity, alpha-cell function, insulin, glucose homeostasis, type 2 diabetes, precision medicine, mathematical model

## Abstract

**Background/Objectives:**

Glucagon is important in the maintenance of glucose homeostasis, with also effects on lipids. In this study, we aimed to apply a recently developed model of glucagon kinetics to determine the sensitivity of glucagon variations (especially, glucagon inhibition) to insulin levels (“alpha-cell insulin sensitivity”), during oral glucose administration.

**Subjects/Methods:**

We studied 50 participants (spanning from normal glucose tolerance to type 2 diabetes) undergoing frequently sampled 5-hr oral glucose tolerance test (OGTT). The alpha-cell insulin sensitivity and the glucagon kinetics were assessed by a mathematical model that we developed previously.

**Results:**

The alpha-cell insulin sensitivity parameter (named S_GLUCA_; “GLUCA”: “glucagon”) was remarkably variable among participants (CV=221%). S_GLUCA_ was found inversely correlated with the mean glycemic values, as well as with 2-hr glycemia of the OGTT. When stratifying participants into two groups (normal glucose tolerance, NGT, *N*=28, and impaired glucose regulation/type 2 diabetes, IGR_T2D, *N*=22), we found that S_GLUCA_ was lower in the latter (1.50 ± 0.50·10^-2^ *vs*. 0.26 ± 0.14·10^-2^ ng·L^-1^_GLUCA_/pmol·L^-1^_INS_, in NGT and IGR_T2D, respectively, p=0.009; “INS”: “insulin”).

**Conclusions:**

The alpha-cell insulin sensitivity is highly variable among subjects, and it is different in groups at different glucose tolerance. This may be relevant for defining personalized treatment schemes, in terms of dietary prescriptions but also for treatments with glucagon-related agents.

## Graphical Abstract

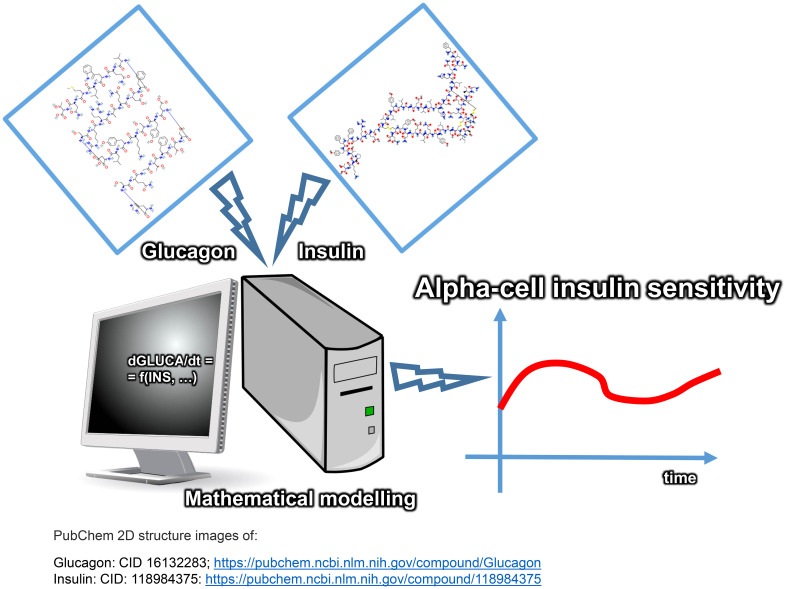

## 1 Introduction

The traditional view of glucagon role in the regulation of glucose homeostasis is that of a key actor as counterregulatory hormone preventing hypoglycemia in fasting conditions, by increasing hepatic glucose production through stimulation of gluconeogenesis and glycogenolysis. Thus, glucagon action is functionally antagonistic to the anabolic action of insulin. On the other side, although it may sound paradoxical, glucagon has been found also able to stimulate rather than suppress insulin secretion, due to the reason that glucagon signals via G protein-coupled receptors (not only the glucagon receptor, GCGR, but also the glucagon-like peptide 1 receptor, GLP-1R) (1). Therefore, these actions of glucagon are similar to those of the glucagon-like peptide 1 (GLP-1), which is processed from the same glucagon precursor, and is well known to potentiate glucose-stimulated insulin secretion. Since beta cells express both GLP-1R and GCGR (1), glucagon can activate both receptors to promote the recruitment and fusion of additional insulin granules from the beta cells, thus potentiating insulin secretion (2). Obviously, this phenomenon only occurs in stimulated conditions, thus ensuring that insulin release is potentiated only in the case of nutrient excess (*i.e.*, in postprandial conditions) (1). However, glucagon exerts other relevant metabolic actions. For instance, it was reported that glucagon has hypolipidemic effects, determining decrease in triglycerides and cholesterol, and increase in free fatty acid oxidation (3–6). Based on these premises, it is not surprising that glucagon plays a remarkable role in the pathophysiology of type 2 diabetes (T2D). In fact, in T2D both elevated plasma glucagon levels at fasting and defective inhibition of glucagon secretion in the postprandial state can be observed, related to an altered insulin suppression of the pancreatic alpha-cell glucagon exocytosis (7, 8). Those abnormalities are often already observable at an early stage of T2D development, and even in impaired glucose tolerance conditions (9). In addition, increased fasting glucagon and delayed glucagon suppression have been shown to go along with insulin resistance even in subjects with normal glucose tolerance (10).

The interest in the study of glucagon, however, is not simply due to its relevance in glucose homeostasis and in the pathophysiology of T2D. Indeed, the study of glucagon has direct clinical implications for the treatment of diabetes. First, in patients with diabetes suffering from severe hypoglycemic events the administration of glucagon is an important therapeutic option (11). Furthermore, different pharmacological agents involving GCGR agonists are under development (12). There are in fact several GCGR/GLP-1R coagonists in development for the treatment of diabetes, obesity, and nonalcoholic steatohepatitis (13). Preclinical evaluation of these agents showed remarkable effects on weight loss and related metabolic improvement. As an example, SAR425899 is a GCGR/GLP-1R coagonist derived from exendin-4 with higher activity at the GLP-1R (14), which provided weight loss in both healthy participants and T2D patients (15). Cotadutide is a GCGR/GLP-1R coagonist derived from oxyntomodulin, with balanced activity at both receptors. LY2933876 is also based on oxyntomodulin, but with action tilted toward GLP-1R (16). Several other GCGR/GLP-1R coagonists are at various stages of development or investigation (17). In addition, it has to be noted that the glucose-dependent insulinotropic polypeptide (GIP) has recently gained renewed interest for the treatment of diabetes due to the clinical success of the GIP/GLP-1 coagonist tirzepatide (18). Accordingly, the potential of GIP receptor (GIPR) agonism in having independent actions enhancing GLP-1R agonism has led the development of GCGR/GLP-1R coagonists toward multi-receptor coagonists that engage GCGR, GLP-1R, and GIPR as well (19). Clinical trials based on a GCGR/GLP-1R/GIPR triagonist, are ongoing (12). Based on this information, it is therefore evident that the interest for the potential role of glucagon in the treatment of diabetes (as well as obesity) is currently outstanding. In addition, with regard to the clinical implications for diabetes treatment, it is worth noting that different dietary regimens have effects on glucagon release and action (20–23). Thus, the study of glucagon kinetics in postprandial conditions is important, and may allow precise assessment of the effects of different nutritional schemes on the main glucometabolic variables.

Recently, we developed a mathematical model for the assessment of glucagon kinetics during an oral glucose tolerance test (OGTT), allowing estimation of glucagon-related parameters in the single individual (24). Specific interest of the model approach is for one parameter with considerable potential for clinical applications, *i.e.*, the sensitivity to glucose-induced insulin release of the glucagon variations (especially, inhibition), which is denoted as alpha-cell insulin sensitivity (24). Notably, it is important to estimate parameters, with direct physiological meaning, in the single subject. Indeed, this may have remarkable potential for personalized treatment strategies, with regard to both lifestyle (nutritional) prescriptions and pharmacological treatments, in the context of precision medicine (25–28). In this study, we aimed to apply our model approach for the assessment of alpha-cell insulin sensitivity and glucagon kinetics in a group of individuals spanning from normal glucose tolerance to overt T2D. To our knowledge, this is the first study assessing in single individuals the alpha-cell insulin sensitivity following oral glucose administration.

## 2 Subjects and methods

### 2.1 Study participants and experimental procedures

The cohort of the present study included 50 adult subjects participating in the CATAMERI study (Catanzaro Metabolic Risk factors), which is an observational study recruiting individuals carrying at least one risk factor among overweight/obesity, hypertension, dyslipidemia, dysglycemia, and family history of T2D (29, 30). In this investigation, we specifically included those subjects showing statistically significant relationship between glucagon and insulin. Independently of the presence of such relationship, we excluded those subjects with end-stage renal disease, chronic gastrointestinal diseases, chronic pancreatitis, history of any malignant disease, high triglycerides (>400 mg/dL), self-reporting high alcohol consumption (>20 g/day), positivity for antibodies to hepatitis C virus or hepatitis B surface antigen.

All included participants underwent, after a 12-hr fast, a 5-hr 75 g OGTT with sampling at fasting and every 30 min following glucose ingestion (*i.e.*, 11 time samples). Glucose was determined by an enzymatic method (Roche, Basel, Switzerland). Insulin was measured with a chemiluminescence-based assay (Immulite, Siemens Healthcare GmbH, Erlangen, Germany), whereas for glucagon a radioimmunoassay kit was used (Millipore Corporation, Billerica, MA), as previously described (31). HbA1c was determined with high performance liquid chromatography using a National Glycohemoglobin Standardization Program (NGSP) certified automated analyzer (Adams HA-8160 HbA1c analyzer, Menarini, Italy). Individuals were classified according to the American Diabetes Association criteria: normal glucose tolerance (NGT), impaired glucose regulation (IGR, as one or more defects among impaired fasting glucose, impaired glucose tolerance, HbA1c-prediabetes), and T2D (32). All participants also underwent anthropometric evaluation; height was measured to the nearest 0.1 cm, while body weight was measured using a calibrated electronic scale to the nearest 0.1 kg.

The study was approved by the Institutional Ethics Committee of the University “Magna Graecia” of Catanzaro (approval code: 2012.63) and written informed consent was obtained from all participants in accordance with the principles of the Declaration of Helsinki.

### 2.2 The model of glucagon kinetics

In this study, we exploited a mathematical model of glucagon kinetics that was previously developed (24). Briefly, the model is based on the hypothesis of the “intra-islet interaction” (33, 34), assuming that variations of glucagon secretion during an OGTT are mainly determined by the glucose-induced insulin secretion levels. The model is composed of two compartments, namely plasma glucagon compartment and remote (from plasma) insulin compartment:


(1) $$\begin{array}{l} \frac{dGluca\left(t\right)}{dt}=-K_{GLUCA}\cdot Gluca\left(t\right)-S_{GLUCA}\left(t\right)\cdot \frac{d\Delta Ins_{remote}\left(t\right)}{dt}\\ Gluca\left(0\right)=Gluca_{b} \end{array}$$

(2) $$\begin{array}{l} \frac{d\Delta Ins_{remote}\left(t\right)}{dt}=-K_{\Delta INSREM}\cdot \Delta Ins_{remote}\left(t\right)+\left(Ins_{plasma}\left(t\right)-Ins_{b}\right)\\ \Delta Ins_{remote}\left(0\right)=0 \end{array}$$

In Equation (1), Gluca(t) (ng·L^-1^) is the glucagon concentration in the plasma compartment, and K_GLUCA_ (min^-1^) represents the glucagon elimination rate from plasma due to clearance operated by liver and kidney. S_GLUCA_(t) (ng·L^-1^ of glucagon divided by pmol·L^-1^ of insulin) is a time-varying parameter expressing the sensitivity of glucagon variations to glucose-induced insulin release during the OGTT (*i.e.*, an alpha-cell insulin sensitivity, which can be assumed as a parameter of alpha-cell function). Specifically, in the first part of the OGTT this parameter represents the sensitivity to insulin of glucagon inhibition, whereas in the last part of the OGTT the parameter represents the sensitivity to insulin of glucagon returning to the basal value. More explicitly, in the first part of the OGTT higher values of S_GLUCA_(t) indicate greater ability of insulin variations to suppress glucagon, while in the later OGTT phase higher values of S_GLUCA_(t) indicate greater effect of insulin variations to determine glucagon return to the basal level. With reference again to Equation (1), Gluca_b_ represents the basal plasma glucagon concentration measured during the test. In Equation (2), ΔIns_remote_(t) (pmol·L^-1^) is the suprabasal insulin concentration in a compartment remote from plasma, which represents a delayed version of suprabasal plasma insulin concentration measured during the test, Ins_plasma_(t) (pmol·L^-1^), with Ins_b_ being its basal value; K_ΔINSREM_ (min^-1^) is the insulin elimination rate from the remote compartment. The model parameters to be estimated in the single individual are: K_GLUCA_, S_GLUCA_(t), K_ΔINSREM_. In this study, we will focus on S_GLUCA_(t), which is the parameter expected to yield the most relevant information for clinical purposes. The code implementing the model is available at the following link: https://github.com/micamoret/GlucagonMathModel.

### 2.3 Calculations and statistical analysis

In addition to the model-derived parameters, further metabolic variables were calculated using glucose and insulin data obtained at fasting and during the OGTT. Insulin resistance was computed at fasting as the HOMA-IR index (35), whereas insulin sensitivity during the OGTT was computed as the PREDIM index (36). Beta-cell function was computed at fasting as Ins_b_/Glu_b_, where Ins_b_ is basal (fasting) insulin and Glu_b_ is basal glucose, and during the OGTT as the Insulinogenic Index, IGI, as well as AUC_INS_/AUC_GLU_, and ΔAUC_INS_/ΔAUC_GLU_, where AUC indicates the area-under-the-curve during the OGTT (37). Variability in parameter/variable values was assessed in terms of coefficient of variation (CV).

Before statistical testing, parameter/variable distributions were tested according to Shapiro-Wilk’s test, and values were transformed (natural logarithm) in case of skewed distributions, unless otherwise specified (see below). Analysis of variance (ANOVA) and Tukey’s honestly significant difference (HSD) *post-hoc* test were used for group comparisons of variables (specifically, for group comparisons of mean S_GLUCA_, *i.e.*, the mean value of S_GLUCA_ calculated individual by individual over the whole OGTT, or, in some cases, over a temporal portion of the OGTT).

Wilcoxon rank-sum test was used to analyze differences between pairs of participants’ groups for variables with skewed distributions and negative values (mainly S_GLUCA_) to bypass the problem of logarithmic transformation of negative values. Difference from zero of such variables was analyzed by one sample Wilcoxon test. Correlation between variables was tested by Spearman rank correlation. Regression plots were also reported for some variables.

Two-sided p-value less than 0.05 was considered statistically significant. Values are reported as mean ± standard error (SE) unless otherwise specified. Statistical analyses were performed in R (version 3.6.3) and contributing packages.

## 3 Results

### 3.1 Main characteristics

**Figure 1:**
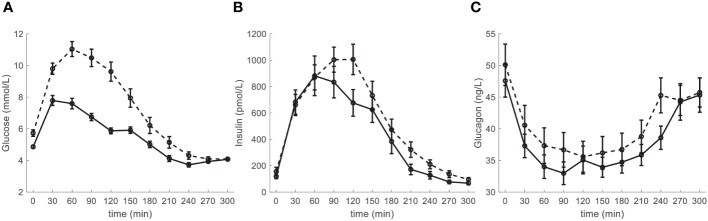
Time course of **(A)** glucose, **(B)** insulin and **(C)** glucagon, in the participants stratified according to glucose tolerance: NGT (normal glucose tolerance): solid line; IGR (impaired glucose regulation (prediabetes)) and T2D (type 2 diabetes) pooled (IGR_T2D): dashed line. Data are reported as mean ± SE.

The main characteristics of the participants are reported in **Table 1**, stratified according to their glucose tolerance (NGT, *N*=28, and IGR and T2D pooled (IGR_T2D), *N*=22). Participants in the IGR_T2D group were slightly older, and tended to be more obese. Glycemic values were as expected higher in IGR_T2D. Insulin tended to be higher in this group (though significant difference was not reached), suggesting still partial compensatory augmentation of insulin secretion to counterbalance hyperglycemia. In contrast, glucagon levels did not show significant difference, despite a tendency to impaired inhibition in IGR_T2D (*i.e.*, tendency to higher values both at fasting and on average). Though cholesterol levels were not different between the two groups, triglycerides were higher in IGR_T2D. Both systolic and diastolic blood pressure values were also higher in IGR_T2D. The time course of glucose, insulin and glucagon in the two groups is reported in **Figure 1**.

**Table 1:** Main characteristics of the participants stratified according to glucose tolerance (NGT: normal glucose tolerance; IGR_T2D: impaired glucose regulation (prediabetes) and type 2 diabetes pooled).

	NGT	IGR_T2D
*N*	28	22
Sex (male/female)	7/21	12/10
Age (years)	35.9 ± 2.2	42.2 ± 2.2 ^*^
BMI (kg/m^2^)	33.0 ± 2.1	37.5 ± 1.9
HbA1c (%)	5.30 ± 0.06	5.97 ± 0.20 ^*^
Fasting glucose (mmol/L)	4.86 ± 0.09	5.73 ± 0.21 ^*^
2-hr glucose (mmol/L)	5.87 ± 0.19	9.61 ± 0.61 ^*^
Mean glucose (mmol/L)	5.65 ± 0.13	7.53 ± 0.32 ^*^
Fasting insulin (pmol/L)	118.5 ± 17.1	155.5 ± 32.3 ^*^
Mean insulin (pmol/L)	505.1 ± 80.0	592.4 ± 69.5
Fasting glucagon (ng/L)	47.6 ± 2.6	50.1 ± 3.3
Mean glucagon (ng/L)	38.3 ± 1.8	41.1 ± 2.4
HDL-cholesterol (mmol/L)	1.30 ± 0.07	1.12 ± 0.07
LDL-cholesterol (mmol/L)	3.15 ± 0.16	3.33 ± 0.23
Triglycerides (mmol/L)	1.13 ± 0.10	1.74 ± 0.18 ^*^
Systolic blood pressure (mmHg)	118.7 ± 2.9	129.1 ± 3.0 ^*^
Diastolic blood pressure (mmHg)	75.9 ± 2.0	82.6 ± 2.2 ^*^

^*^p<0.05 IGR_T2D *vs.* NGT. BMI, body mass index. Continuous variables are reported as mean ± SE. Mean glucose, insulin, glucagon refer to the mean values during the oral glucose tolerance test.

### 3.2 Model analysis and alpha-cell function assessment

**Figure 2:**
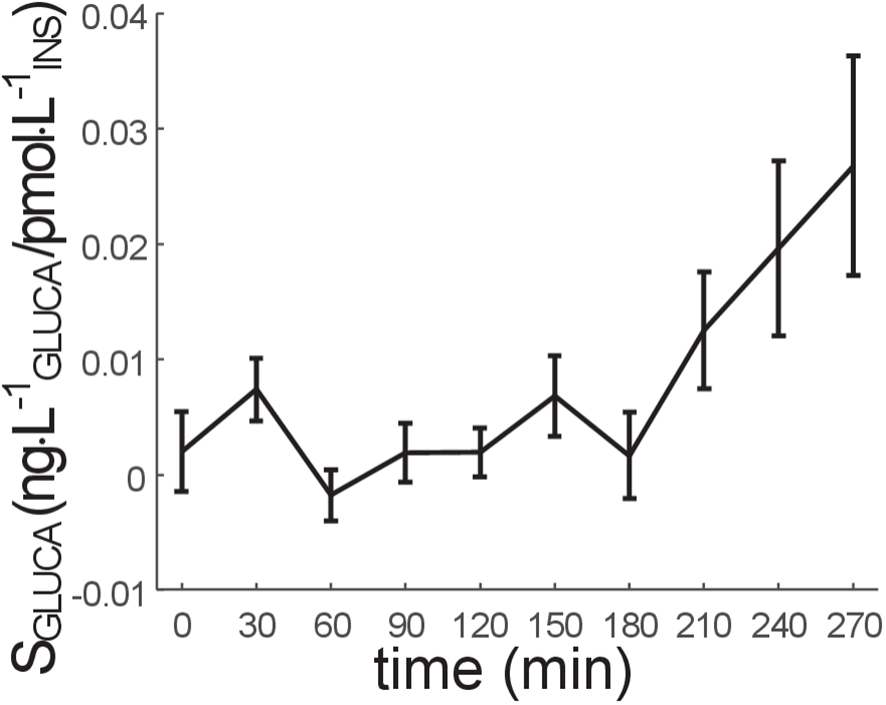
Time course of S_GLUCA_ parameter (alpha-cell insulin sensitivity) (mean ± SE). S_GLUCA_ value at the last time sample (300 min) is not reported, since it is not calculated (it is hypothesized that S_GLUCA_ at 300 min is equal to S_GLUCA_ at 270 min).

**Figure 3:**
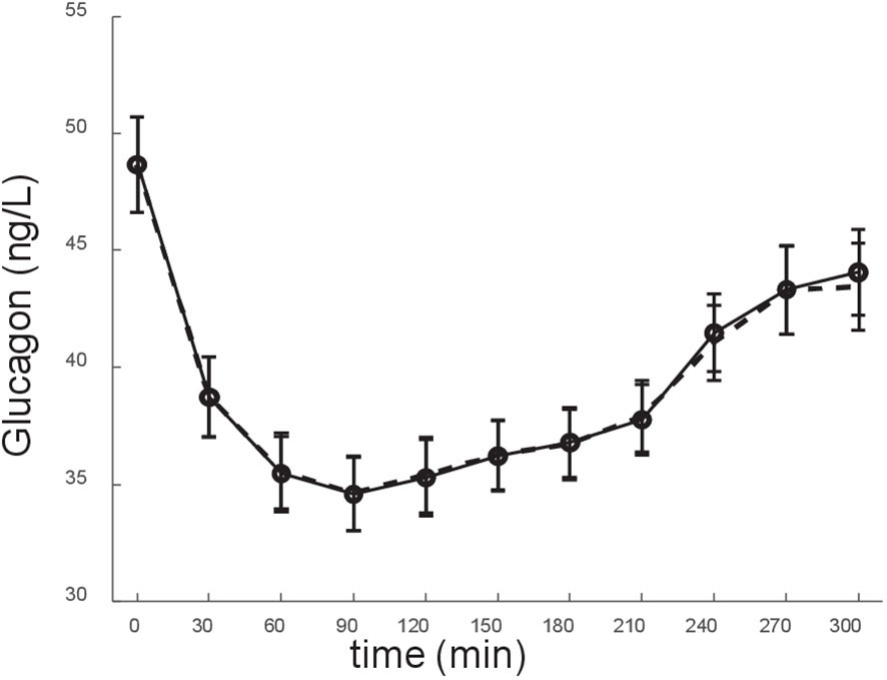
Model fit of the experimental data (glucagon data). Experimental data: solid line; model fit: dashed line. Data are reported as mean ± SE.

The time course of S_GLUCA_ in all participants is reported in **Figure 2**, whereas the corresponding model fit performance is reported in **Figure 3**. The S_GLUCA_ mean value during the OGTT was 0.95 ± 0.30·10^-2^ ng·L^-1^_GLUCA_/pmol·L^-1^_INS_ (in the S_GLUCA_ units, “GLUCA” and “INS” suffix stands for “glucagon” and “insulin”, respectively). Mean S_GLUCA_ was remarkably variable among participants (CV=221%), likely due to the heterogeneity of the studied participants in terms of glucose tolerance. As regards the other model parameters, K_GLUCA_ and K_ΔINSREM_ were 2.70 ± 0.71·10^-3^ min^-1^ and 9.33 ± 0.81·10^-2^ min^-1^, respectively.

### 3.3 Relationships between model-derived alpha-cell function and other metabolic parameters

**Figure 4:**
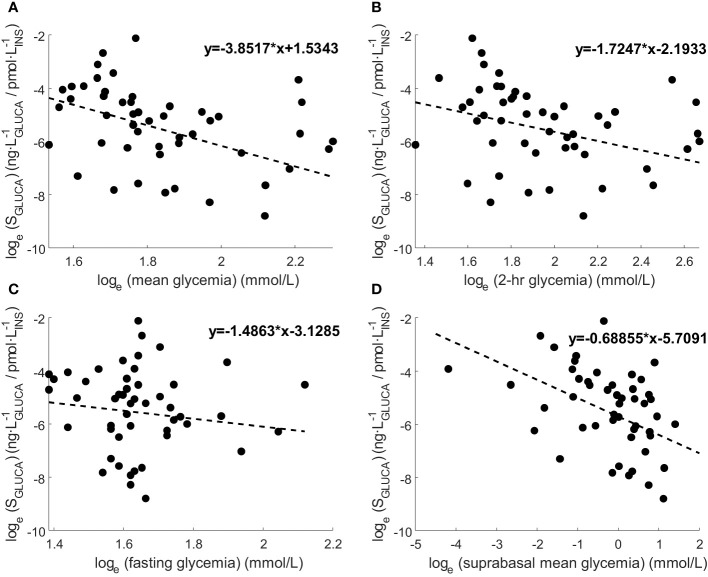
Regression plots of S_GLUCA_ and **(A)** mean glycemia, **(B)** 2-hr glycemia, **(C)** fasting glycemia and **(D)** mean suprabasal glycemia. Variable values are transformed according to natural logarithm. Regression line equation is also reported. Correlation results according to Spearman rank correlation (rho and p-value) are reported in Section 3.3 (significant for mean glycemia, 2-hr glycemia, mean suprabasal glycemia, not significant for fasting glycemia).

We analyzed the relationships of individual mean S_GLUCA_ (*i.e.*, mean S_GLUCA_ value over the OGTT of one subject) and different metabolic parameters. We found that mean S_GLUCA_ was inversely correlated with the mean glycemic values (rho=-0.30, p=0.03), as well as with 2-hr glycemia (rho=-0.33, p=0.02), but not with fasting glycemia (p=0.80). Thus, when assessing the relationship of S_GLUCA_ with the suprabasal glycemic component, correlation became somehow stronger (rho=-0.40, p=0.004). Regression plots of S_GLUCA_ and such glycemic levels are reported in **Figure 4**.

When analyzing possible correlation with insulin sensitivity/resistance, S_GLUCA_ was not correlated either with HOMA-IR or with PREDIM (p≥0.57). Similarly, we did not find correlation with beta-cell function indices, as assessed by Ins_b_/Glu_b_, IGI, AUC_INS_/AUC_GLU_ and ΔAUC_INS_/ΔAUC_GLU_ (p≥0.31).

### 3.4 Model analysis and alpha-cell function in participants stratified according to glucose tolerance and other criteria

**Figure 5:**
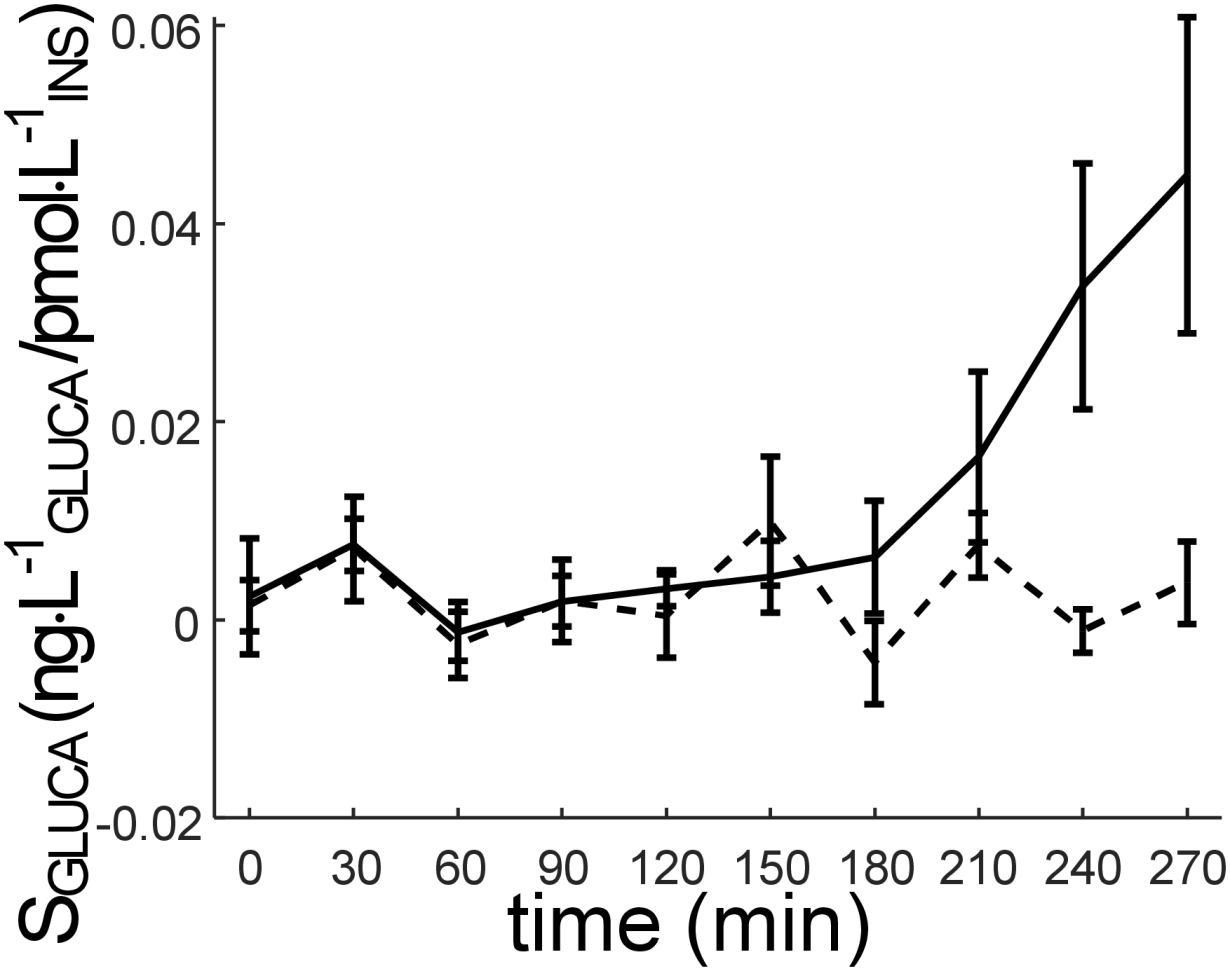
Time course of S_GLUCA_ parameter (alpha-cell insulin sensitivity) (mean ± SE) in the participants stratified according to glucose tolerance: NGT (normal glucose tolerance): solid line; IGR (impaired glucose regulation (prediabetes)) and T2D (type 2 diabetes) pooled (IGR_T2D): dashed line. S_GLUCA_ value at the last time sample (300 min) is not reported, since it is not calculated (it is hypothesized that S_GLUCA_ at 300 min is equal to S_GLUCA_ at 270 min).

We analyzed S_GLUCA_ in participants stratified according to their glucose tolerance, *i.e.*, NGT and IGR_T2D, as previously defined. In NGT, the mean S_GLUCA_ (mean value of S_GLUCA_ during the OGTT calculated in each individual) was 1.50 ± 0.50·10^-2^ ng·L^-1^_GLUCA_/pmol·L^-1^_INS_. In IGR_T2D, the same parameter was 0.26 ± 0.14·10^-2^ ng·L^-1^_GLUCA_/pmol·L^-1^_INS_, and a significant difference was observed between the two groups (p=0.009). The time course of S_GLUCA_ in NGT and IGR_T2D is reported in **Figure 5**.

Visual inspection of **Figure 5** suggests that, on average, the main difference in S_GLUCA_ between NGT and IGR_T2D may occur in the late part of the OGTT, *i.e.*, from 180 min onwards (phase of glucagon return towards the basal value). However, when comparing S_GLUCA_ between NGT and IGR_T2D limited to the 180-300 min time interval, significant difference was confirmed, but with virtually the same p-value as found over the whole time interval (p=0.009), likely due to the higher variability in such late interval (larger SE bars: see **Figure 5**).

We then grouped the participants according to sex (males: *N*=23; females: *N*=27), obesity condition (BMI≥30 kg/m^2^: *N*=32, non-obese: *N*=18) and age (elderly: ≥50 years: *N*=8; young: *N*=42). However, we found no difference in any of the comparisons according to the indicated factors (p≥0.65).

With regard to the other model-derived parameters, K_GLUCA_ was 1.91 ± 0.66·10^-3^ min^-1^ in NGT and 3.70 ± 1.25·10^-3^ min^-1^ in IGR_T2D, but significant difference was not reached between the two groups. Similarly, no significant difference was found for K_GLUCA_ in any other comparison with participants grouped according to sex, obesity condition and age. K_ΔINSREM_ was 11.65 ± 1.11·10^-2^ min^-1^ in NGT and 6.37 ± 0.94·10^-2^ min^-1^ in IGR_T2D, with difference between groups found significant (p=0.0007). In contrast, no significant difference was found for K_ΔINSREM_ in any other group comparison.

### 3.5 Pharmacological treatment

At the time of the study, some of the participants were under pharmacological treatment with one or more agents of different categories, mainly to treat hypertension (ace inhibitors, sartans, calcium antagonists, alpha or beta blockers, diuretics), but also hyperlipidemia (statins, fibrates), and hyperglycemia as well (metformin). Interestingly, when comparing the mean S_GLUCA_ between participants under some pharmacological treatment (*N*=13) and those without any treatment (*N*=37), we observed different (somewhat higher) values in the former group (p=0.011), suggesting a possible effect of pharmacological treatments on alpha-cell function. However, this aspect certainly needs to be elucidated in future studies, with higher number of participants under treatment.

## 4 Discussion

### 4.1 Summary of study aims and main findings

In this study, we applied a model of glucagon kinetics for the individual assessment of the pancreatic alpha-cell sensitivity to insulin, leading to glucagon variations (initial inhibition and then return to basal), which we defined shortly as alpha-cell function. We studied participants with heterogeneous glucose tolerance, ranging from normal glucose tolerance to diabetes. Our modelling approach was previously developed and validated on average patients’ data derived from the literature, and on virtual, in-silico generated patients (24). The present study is the first application of our model to real individual patients’ data. Most importantly, to our knowledge this is the first study providing an assessment of the alpha-cell sensitivity to insulin following oral glucose administration in single individuals.

Our main findings were that such alpha-cell insulin sensitivity (the S_GLUCA_ model parameter) is markedly heterogeneous among individuals, but nonetheless as compared to the participants with normal glucose tolerance it was lower in the participants with some degree of dysglycemia (*i.e.*, in the group with impaired glucose regulation or type 2 diabetes). This suggests that the regulation of glucagon levels (at least, the portion of it that is affected by the insulin action) can be impaired in people with abnormal glucose tolerance. This was also corroborated by a significant (though not strong) inverse correlation between S_GLUCA_ and glycemia. On the other hand, it is worth noting that the difference in S_GLUCA_ between the participants with normal glucose tolerance and those with dysglycemia appears more evident in the last part of the OGTT, when glucagon tends to return to the basal value.

### 4.2 Possible determinants of glucagon kinetics and considerations in the general context of glucose homeostasis

We also investigated possible relationships between alpha-cell function (S_GLUCA_) and beta-cell function, as well as insulin sensitivity and resistance. At first sight, this lack of relationship with some of the main glucometabolic factors may be surprising and may downplay the insulin-related glucagon role in the regulation of glucose homeostasis. However, at deeper thought, our findings may suggest some considerations. Indeed, the effectiveness of the mechanism of glucagon regulation following a glucose load may be an independent metabolic factor, thus possibly unrelated to others, contributing to glucose homeostasis. This view is plausible and in line with the indications of several studies suggesting a plethora of physiological mechanisms contributing to glucose homeostasis, such as those designed as the “ominous octet” (including glucagon action) (38). On the other side, it has to be acknowledged that in our study, we only assessed the ability of insulin in regulating glucagon levels, but the latter is likely not negligibly affected by other factors. In fact, there is currently a remarkable interest in the study of the determinants of glucagon behavior (mainly, secretion, inhibition, kinetics and action), since it has become progressively more evident that many factors may be involved. Some review studies reported indeed that multiple factors affect glucagon behavior, among which nutrients, autocrine, paracrine, endocrine and neural factors (39–41). As an example, glucose may affect glucagon through different independent mechanisms, and may either stimulate or suppress glucagon secretion (41). The beta cell determines glucagon inhibition not only through insulin, but also amylin (40), as well as through some neurotransmitters (serotonin, and GABA) (39). The delta cell suppresses glucagon through somatostatin (39–41). Curiously, incretin hormones have opposite effects on glucagon, since GLP-1 may determine inhibition, whereas depending on the glycemic levels GIP may determine stimulation (40). Furthermore, some recent studies showed that other factors affect glucagon secretion, such as the insulin-like growth factor-1 (IGF-1) (31), the high-density lipoprotein (HDL), and the apolipoprotein A-1 (ApoA-1) (42). In addition, amino acids may affect both glucagon stimulation and suppression, depending on the specific amino acid (43). Environmental factors, both biochemical and physical, can also affect glucagon (44). The fact that several factors may be significant determinants of glucagon levels appears also consistent with the findings of one of our previous studies on glucagon involving the different macronutrients (*i.e.*, glucose, proteins and fat) (45). In that study, we found that the relationship between glucagon and insulin is more frequent than that between glucagon and glucose, in agreement with the present study (not shown). Of note, in that previous study (45) this was observed after ingestion of both glucose and the other macronutrients or with the macronutrients combined. On the other side, the glucagon-insulin relationship, even when significant, was sometimes weak.

### 4.3 Clinical potential of the study

What clinical implications may the present study have? In our previous study mentioned above (45), we observed that the macronutrients differently affect the glucagon release and inhibition. This is consistent with the findings of some studies showing the effects on glucagon of different dietary regimes (20–23). On the other side, given the role of glucagon as one of the determinants of glucose homeostasis, it has to be emphasized that glucagon release and inhibition may be relevant factors, among others, for preventing deterioration of the metabolic condition and hence hampering the onset of type 2 diabetes (or avoiding further metabolic derangement in people already with the disease). The present study makes a step forward in the assessment of the effects of nutrients ingestion (though currently limited to glucose) on glucagon release and inhibition, since it allows a quantitative evaluation of such effects through the model parameter (S_GLUCA_) describing the sensitivity of glucagon variations to the insulin levels. This appears of potential interest when prescribing personalized nutritional interventions specifically tailored for the single patient, in the context of nutrition therapy (46–48) and precision nutrition in diabetes (49–51). In addition, it has to be noted that a refined, quantitative assessment of the insulin effects on glucagon may be important for personalized treatment plans with some modern therapeutic agents, such as the glucagon/GLP-1 receptor coagonists, or even glucagon/GLP-1/GIP triagonists, as previously indicated (11–18).

### 4.4 The choice of the “minimal-model” approach and related implications

As discussed above, a wide battery of factors affects glucagon release and inhibition, and hence glucagon kinetics and action. However, in our model approach, we considered only insulin. This is due to the reason that we aimed to develop a “minimal model”, which could then be used for the assessment of the model parameters (especially, S_GLUCA_) in the single individual, thus with potential for real, clinical applications. Developing a model requiring several inputs acting as glucagon determinants would make the model practically unusable in the clinical context, since the higher are the requirements in terms of experimental data, the lower is the chance of having such data collected in the clinical practice. Of note, the minimal model approach has a long-standing tradition in the study of glucose metabolism (52–54), and our group has recently opted for such approach even in other recent studies (55). Thus, in our model approach for the study of glucagon, we pursued the minimal model tradition, and hence we opted for a single determinant of glucagon levels. Our choice was for insulin rather than glucose because, as already discussed, in our experience insulin is typically more related than glucose to glucagon. On the other hand, since both insulin and glucose are often available in the clinical context, our plans for future studies in the field include the development of a model version with both of them as glucagon determinants. It has however to be acknowledged that in this case the model applicability in the clinical context may be challenging, since the uncertainty in model parameters estimation may increase in case of similar time course of insulin and glucose (*a posteriori* identifiability).

The reader may wonder whether these “minimal models” have real chances to enter the clinical practice. As a fact, we do not expect these models shortly becoming common in the clinical routine, but they are applicable, and indeed already applied, in clinical trials, especially when the size of the cohort is not extremely high and hence intensive phenotyping of the participants is possible. In addition, in our opinion, a clear trend is delineated towards progressively increasing use of advanced data analysis methodologies (including mathematical modelling) in the clinical practice, since precision medicine recommendations emphasize the need for individual, personalized diagnostics, treatment and general care (25–28). This certainly takes advantage of refined patient’s data analysis allowed by mathematical models, as also outlined in studies focused on precision medicine in the metabolic field (56, 57). Of note, this process will also be promoted by the advancements in measurement science. Immunoassays are already available for concomitant measures of different variables of interest in metabolism (as an example, insulin and glucagon, plus GLP-1, possibly), and in the near future metabolic assay kits will allow measurement of several variables, with tendency towards decreasing prices.

Some further considerations are pertinent regarding mathematical modelling. In fact, some investigators may be skeptical about the actual ability of modelling to effectively disclose information not provided by simpler approaches, such as easy algebraic formulas (sometimes called “empirical indices”, *i.e.*, empirically found as useful, but with no physiologic background). In our opinion, it is correct that in many cases the empirical indices yield information comparable to that of more complex, model-based approaches. However, mathematical models are often less prone to typical limitations of the empirical indices (such as the presence of outliers) and finally result in improved sensitivity, *i.e.*, higher ability in disclosing subtle differences among groups. This was clearly observed in the present study. Indeed, since our model parameter S_GLUCA_ represents the ability of insulin to promote glucagon variations (mainly, inhibition, and then return towards basal values), one may speculate that such ability could be assessed by a simpler, empirical index, based on the ratio between glucagon and insulin during the OGTT. However, when considering the ratio of the area-under-the-curve of glucagon to that of insulin (AUC_GLUCA_/AUC_INS_), we found no difference between the participants’ groups at different glucose tolerance, in contrast with S_GLUCA_; the same held when suprabasal glucagon and insulin components were considered, *i.e.*, ΔAUC_GLUCA_/ΔAUC_INS_ (details not shown). Of note, in previous studies we already observed this greater ability of the model-based parameters, compared to empirical indices with similar interpretation, in disclosing subtle differences among groups, possibly in agreement with reference parameters obtained by complex experiments (58–61).

### 4.5 Study limitations

This study has some limitations. First, in the mathematical model we used plasma insulin, at difference with the original study (concerning the model development) where we used plasma C-peptide (24), because in this study C-peptide was not available for all participants, and hence only 28 subjects were present with significant relationship between glucagon and C-peptide. This suggested using insulin rather than C-peptide, thus having 50 subjects rather than 28 only to be analyzed. On the other hand, it has to be acknowledged that that plasma C-peptide may be better representative of insulin acting on glucagon at the level of the pancreatic islet. Indeed, plasma C-peptide is considered better marker than plasma insulin of the insulin secretion at the pancreatic level, since C-peptide is secreted equimolarly with insulin, but it does not undergo partial degradation in the liver, which may be quite variable among individuals (62). Nonetheless, our model performed appropriately even with the use of plasma insulin rather than plasma C-peptide, as mirrored by the satisfactory model fit of the experimental data (see **Figure 3**). At any rate, we also run the model over the plasma insulin rather than C-peptide data, which were reported in the article that we exploited in our original study on the glucagon model (24). We found that the model, even with insulin, confirmed its satisfactory performance both in terms of model fit and of reliability in model parameter values (details not shown). Another limitation of the study was the number of participants analyzed with our model approach. On the other side, the size of the cohort was reasonable in relation to the long and frequently sampled OGTT performed in all participants (11 time samples over 5 hours), and considering the wide battery of measured variables, including some not commonly measured in metabolic studies (especially, fetuin-A, interleukin-6, interleukin 1-beta, though not object of the present study). Of note, data from OGTT with similar duration and frequent sampling are rare (even rarer the glucagon data, in these conditions), and this is another merit of our study. In addition, despite the limited sample size of our study, we were able to disclose significant differences in the main model parameter (S_GLUCA_) between the groups at different glucose tolerance condition. This emphasizes the potential of our model approach for possible clinical applications. Finally, it also has to be acknowledged that in this study we included only subjects showing significant relationship between glucagon and insulin (50 subjects). Indeed, the CATAMERI cohort included a total of 126 subjects (with glucagon and insulin measurement available), but 76 subjects did not meet the inclusion criteria of this study related to the existence of a significant relationship between glucagon and insulin (see “Study participants and experimental procedures” section). Of note, the fact that only a portion of the subjects of the total cohort satisfies the criterion of significant glucagon-insulin relationship (with such portion being less than half of the total cohort) appears consistent with what already observed in previous studies (45). On the other hand, as regards this issue, we can claim that the model may be also applied to data where the glucagon-insulin relation condition is not met. In fact, the model would like provide reliable results from a computational point of view. However, results may be not completely meaningful from the physiological/clinical point of view, since in cases where glucagon and insulin are unrelated the insulin action on glucagon may be of limited relevance, and hence the model parameters (especially S_GLUCA_) may be not reliable. For this reason, we opted for a conservative approach, thus selecting only those cases where the glucagon-insulin relation was observed. Another limitation of the study was that we pooled in a single group the subjects with impaired glucose regulation and with type 2 diabetes (IGR_T2D group, *N*=22). In this group, only six subjects had type 2 diabetes, thus we assumed that the most reasonable option for best statistical analysis reliability was to study them grouped to the subjects with impaired glucose regulation. Future studies on glucagon kinetics may therefore focus on type 2 diabetes patients separately.

## 5 Conclusions

In conclusion, in this study we have analyzed data from a long (5-hr) OGTT performed in subjects with glucose tolerance ranging from normal to severely impaired (overt diabetes). By applying to the OGTT data a mathematical model of glucagon kinetics, we have individually assessed some parameters of direct physiological interpretation. The most relevant model parameter was the sensitivity of the glucagon variations to the insulin levels during the OGTT (especially, glucagon inhibition, in the first hours of the test). This parameter can be denoted as alpha-cell insulin sensitivity and assumed as an indication of alpha-cell function. We found that such parameter was lower in the subjects with impaired glucose regulation or diabetes than in those with normal glucose tolerance. This may be of relevance for personalized nutritional interventions, as well as, possibly, pharmacological interventions for hyperglycemic conditions.

## Data availability statement

The raw data supporting the conclusions of this article will be made available by the authors, without undue reservation.

## Ethics statement

The studies involving humans were approved by Institutional Ethics Committee of the University “Magna Graecia” of Catanzaro (approval code: 2012.63). The studies were conducted in accordance with the local legislation and institutional requirements. The participants provided their written informed consent to participate in this study.

## Author contributions

FA: Data curation, Funding acquisition, Resources, Writing – review & editing. EM: Data curation, Resources, Writing – review & editing. MR: Data curation, Resources, Writing – review & editing. BS: Data curation, Resources, Writing – review & editing. MM: Data curation, Resources, Writing – review & editing. GiM: Data curation, Resources, Writing – review & editing. CG: Data curation, Resources, Writing – review & editing. GaM: Data curation, Investigation, Resources, Writing – review & editing. AT: Data curation, Investigation, Resources, Writing – original draft.
